# Serum Thyroid-Stimulating Hormone Levels and Body Mass Index Percentiles in Children with Primary Hypothyroidism on Levothyroxine Replacement

**DOI:** 10.4274/jcrpe.3661

**Published:** 2017-12-15

**Authors:** Asma Shaoba, Sanjib Basu, Stelios Mantis, Carla Minutti

**Affiliations:** 1 RUSH University Graduate College, Masters in Clinical Research Program, Chicago, Illinois, USA; 2 RUSH University Graduate College, Department of Preventive Medicine, Chicago, Illinois, USA; 3 RUSH University Medical Center, Department of Pediatrics, Division of Pediatric Endocrinology, Chicago, Illinois, USA

**Keywords:** Thyroid-stimulating hormone, hypothyroidism, pediatric, children, obesity, body mass index, euthyroid

## Abstract

**Objective:**

To determine the association, if any, between thyroid-stimulating hormone (TSH) levels and body mass index (BMI) percentiles in children with primary hypothyroidism who are chemically euthyroid and on treatment with levothyroxine.

**Methods:**

This retrospective cross-sectional study consisted of a review of medical records from RUSH Medical Center and Stroger Hospital, Chicago, USA of children with primary hypothyroidism who were seen in the clinic from 2008 to 2014 and who were chemically euthyroid and on treatment with levothyroxine for at least 6 months. The patients were divided into two groups based on their TSH levels (0.34-<2.5 mIU/L and ≥2.5-5.6 mIU/L). The data were analyzed by Spearman rank correlation, linear regression, cross tabulation and chi-square, Mann-Whitney U test, and Kruskal-Wallis test.

**Results:**

One hundred and forty-six children were included, of which 26% were obese (BMI ≥95%), 21.9% overweight (BMI ≥85-<95%), and 52.1% of a healthy weight (BMI ≥5-<85%). There was a significant positive correlation between TSH and BMI percentiles (r=0.274, p=0.001) and a significant negative correlation between TSH and serum free T4 (r=-0.259, p=0.002). In the lower TSH group, 68.4% of the children had a healthy weight, while the percentage of obese children was 60.5% in the upper TSH group (p=0.012).

**Conclusion:**

In children diagnosed with primary hypothyroidism who are chemically euthyroid on treatment with levothyroxine, there is a positive association between higher TSH levels and higher BMI percentiles. However, it is difficult to establish if the higher TSH levels are a direct cause or a consequence of the obesity. Further studies are needed to establish causation beyond significant association.

**What is already known on this topic?**

There have been studies in adults that show that higher thyroid-stimulating hormone (TSH) levels are associated with higher body mass index (BMIs). No studies done in children.

**What this study adds?**

It raises the question on whether we should treat pediatric patients with hypothyroidism aiming to keep TSH in the lower end of normal in an effort to improve their BMI.

## INTRODUCTION

Overt hypothyroidism may be associated with changes in energy expenditure and gain in body weight. Most patients stop gaining or lose weight after beginning treatment with levothyroxine and restoration of a biochemically euthyroid state. Weight gain in hypothyroid patients is related partly to myxedema, but this fluid frequently disappears after one to four weeks of treatment with levothyroxine (1,2).

On the other hand, serum thyroid-stimulating hormone (TSH) levels are often elevated in obese children and adults (3,4,5,6,7,8,9,10,11). The mechanism underlying this finding is not yet fully understood. The hyperthyrotropinemia appears to be a result rather than the root of the obesity, but the question as to whether high TSH levels contribute to difficulties with weight loss in obese patients remains unanswered.

TSH has wide normal ranges, but most laboratories use values of about 4.5 to 5.0 mIU/L as the upper limit of what is considered normal range. Approximately 95% of healthy Americans have serum TSH levels between 0.45 and 4.12 mIU/L. According to the Third National Health and Nutrition Examination Survey, for individuals who do not suffer from thyroid disease, the upper normal limit of serum TSH levels is 4.5 mIU/L (12).

Recent attention has focused on the potential relationship between minor abnormalities of thyroid function and changes in body weight in euthyroid subjects. To investigate whether there is a relationship between serum TSH levels and body mass index (BMI) percentiles in children with primary hypothyroidism who are chemically euthyroid and on treatment with levothyroxine, we retrospectively studied the charts of 146 children.

## METHODS

We conducted a retrospective, cross-sectional study that consisted of chart reviews of all cases from 2008 to 2014 of children aged between 6 months and 21 years with a diagnosis of primary hypothyroidism who were on treatment with levothyroxine for at least 6 months and chemically euthyroid. The study population consisted of patients of RUSH University Medical Center (RUMC) and Stroger Hospital (SH) Pediatric Endocrinology Outpatient Clinics (Chicago, IL, USA). One patient who was on nature-thyroid therapy (animal extract thyroid preparation) was also included. Subjects were identified using International Classification of Disease-9 codes. We analyzed the correlation between serum TSH levels and BMI percentiles.

Children with other endocrinopathies or other medical conditions that may affect weight, such as diabetes mellitus, celiac disease and pregnancy, those with abnormal TSH levels, and those who were on any medication that may alter serum TSH levels were excluded. We also excluded eight patients whose BMI and weight for length (W/L) percentiles were less than the 5%.

We enrolled 146 patients with primary hypothyroidism who were chemically euthyroid and met the inclusion criteria. Our study was approved by the Institutional Review Board of the RUMC and SH (approval number: 14062603-IRB01).

### Anthropometric Measurements

Growth and thyroid function were assessed at each clinic visit every three months or bi-annually. Weight was measured with the subject wearing light clothing with his or her shoes off. Height was measured using a wall-mounted, fixed Harpender stadiometer. Recumbent length was measured using an infantometer in children who were two years of age or younger. BMI was calculated by dividing weight (kg) by height square (meter square) and plotted on the age- and gender-specific Centers for Disease Control (CDC) and National Center for Health Statistics BMI growth charts to obtain a percentile ranking. Weight, height, BMI, and W/L percentiles were calculated using CDC charts for girls and boys aged 2 to 20 years and <6 months. In children aged 2 to 18 years, obesity was defined as a BMI ≥95^th^ percentile for age and gender. For this age group, overweight was defined as a BMI percentile of ≥85%-<95%, and healthy weight as a BMI percentile of ≥5%-<85% (13,14). In children aged less than 2 years, it is appropriate to use weight for recumbent length (W/L) percentile to evaluate their weight relative to linear growth (15,16). The term “overweight”, rather than “obese” is used to describe these young children. For this population, overweight is defined as a W/L percentile of ≥95^th^.

Euthyroid status was defined as having a TSH level of 0.35-4.94 mIU/L at RUMC and 0.34-5.60 mIU/L at SH regardless of free T4 levels. The normal range of free T4 is 0.7-1.5 ng/dL at RUMC and 0.58-1.64 ng/dL at SH. Serum TSH and free T4 at RUMC were determined by two-site chemiluminescent enzyme micro particle immune assay (Architect, Abbott Diagnostics, U.S.). They were also determined at SH by two-site chemiluminescent enzyme microparticle immune assay (DXI, Beckman Coulter, U.S.).

To estimate the proportions of healthy weight, overweight, and obese patients within each TSH group, subjects were divided into two groups: those with a lower TSH (<2.5 mIU/L) and those with a higher TSH group (≥2.5 mIU/L). In addition, the subjects were divided according to category of BMI or W/L into healthy weight, overweight, and obese groups. Measurement obtained at the most recent visit were used in these calculations. Demographic (age, gender, and ethnicity) and clinical details were collected during routine, out-patient care.

### Statistical Analysis

Data were recorded in Microsoft Excel 2010 and analyzed using SPSS version 18.0 software (SPSS Inc., U.S.). The correlations between various variables were assessed by the nonparametric Spearman rank correlation. The Mann-Whitney test was used for comparison between two groups, and the Kruskal-Wallis test was used for comparison among larger groups. All reported p-values are two sided. A p-value ≤0.05 was considered as statistically significant.

## RESULTS

Anthropometric characteristics of the subjects are shown in Table 1. After exclusion of subjects who did not meet the inclusion criteria, a total of 327 participants were investigated, and 146 were eligible for these analyses. Forty-six boys (32%) and 100 girls (68%) with primary hypothyroidism who were chemically euthyroid were enrolled. Patients were divided according to their BMI or W/L percentiles (%). Thus 26% of the study group consisted of obese patients (≥95%), 21.9% of overweight patients (BMI ≥85 but less than 95%) and 52.1% of healthy weight patients (BMI ≥5%-<85%).

The median [interquartile range (IQR)] of BMI and W/L percentiles were 82.25% (41.45%), while the median (IQR) of TSH levels was 2.11 mIU/L (2.15 mIU/L) (Table 1). The median TSH was 1.73 mIU/L for boys, while it was 2.35 mIU/L for girls, and the median BMI and W/L percentile values were 71.06 % in boys and 87.08 % in girls (Table 2). The correlation between serum TSH and BMI percentiles was strongly significant in girls (r=0.330, p=0.001), while in boys, this correlation was only marginally significant (r=0.250, p=0.094).

There was a significant positive correlation between serum TSH and BMI or W/L percentiles (r=0.274, p=0.001) and a significant negative correlation between BMI or W/L percentiles and free T4 (r=-0.259, p=0.002).

A multivariate linear regression analysis for the association between serum TSH levels and different variables is shown in Table 3. In this analysis, we used BMI or W/L expressed in standard deviation scores (SDS) instead of percentiles. This analysis revealed that only BMI contributed significantly to the variance of TSH. This association was slightly attenuated after additional adjustments for age, gender, dose of levothyroxine, ethnicity, and body surface area (BSA) [p=0.013, 95% confidence interval (CI)=0.079-0.642]. A multivariate linear regression analysis for the association between serum free T4 and variables revealed that BMI, age, and ethnicity contributed significantly to the variance of serum free T4. In addition, the association between serum free T4 and BMI was slightly attenuated after adjustments for age, gender, dose of levothyroxine, ethnicity, and BSA (p=0.005, 95% CI=-0.0.122- -0.022).

**Figure 1:**
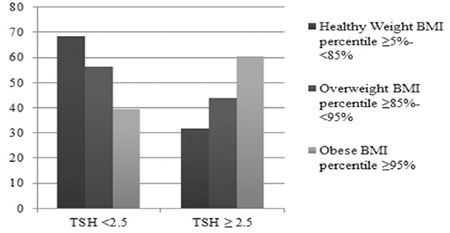
Body mass index percentiles according to thyroid-stimulating hormone (TSH) levels, TSH ≥ than 2.5 mIU/L and < than 2.5 mIU/L BMI: body mass index, TSH: thyroid-stimulating hormone

When subjects were divided according to category of TSH into two TSH groups (< than 2.5 mIU/L and ≥ than 2.5 mIU/L), the percentage of healthy weight subjects in the lower TSH group was 68.4%, while the percentage of obese subjects in the upper TSH group was 60.5% (p=0.012) (Figure 1).

**Figure 2:**
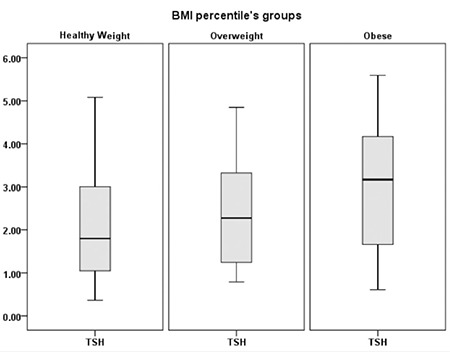
Serum thyroid-stimulating hormone (illustrated as median ± interquartile range) in 146 hypothyroid children divided into healthy weight body mass index (BMI) percentile ≥5-<85%, overweight BMI percentile ≥85-<95%, and obese BMI percentile ≥95% BMI: body mass index, TSH: thyroid-stimulating hormone

**Figure 3:**
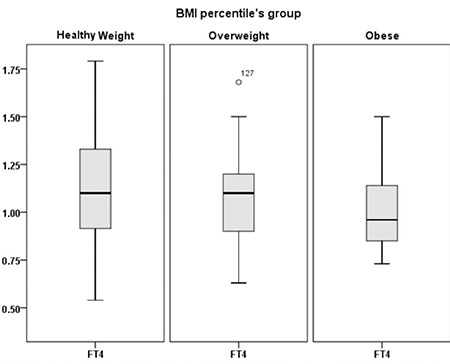
Serum free T4 (illustrated as median ± interquartile range) in 146 hypothyroid children divided into healthy weight body mass index (BMI) percentile ≥5-<85%, overweight BMI percentile ≥85-<95%, and obese BMI percentile ≥95 BMI: body mass index, fT4: free thyroxine

Furthermore, when the subjects were divided according to category of BMI or W/L percentiles into healthy weight, overweight, and obese groups, there was a significant difference in the median serum TSH levels between these groups, which were 1.8 mIU/L in the healthy weight group, 2.3 mIU/L in the overweight group, and 3.2 mIU/L in the obese group (p=0.006) (Figure 2). We also found a significant statistical difference in the median serum free T4 in these groups. It was 1.10 ng/dL in the heathy weight group, 1.10 ng/dL in the overweight group, and 0.96 ng/dL in the obese group (p=0.027) (Figure 3).

## DISCUSSION

In the present study of 146 children with primary hypothyroidism who were chemically euthyroid and on treatment with levothyroxine, we found an association between TSH, free T4, and BMI percentiles when we considered them as continuous variables. This association was also significant when we divided TSH into 2 categories. There was a significant difference in the proportion of healthy weight (BMI percentile ≥5-<85%) and obese subjects (BMI percentile ≥95%) within the two TSH groups (p=0.012). Linear regression revealed that BMI (expressed as SDS) contributed significantly to the variance of TSH. The high proportion of healthy weight patients within the lower TSH group as well as the patients with low serum TSH levels had low BMI values. Thus we venture to suggest that adjusting TSH to the lower end of normal range may be of benefit for weight reduction.

The association between serum TSH and BMI has been addressed by some researchers in both pediatric and adult patients, but the results are controversial. Most studies have been conducted in populations without thyroid disease, but several studies of these studies have reported a positive association between TSH and BMI, (3,4,5,6,7,8,9,10,11) and showed that even small changes in serum TSH levels can affect BMI. Some studies have shown a positive association between higher BMI and serum TSH level in euthyroid adults (17,18,19). Higher TSH levels may contribute to the difficulty of weight loss (5,20). Our patients with higher BMIs had higher TSH levels, although these were within the euthyroid range.

The mechanisms of the association between serum TSH and BMI have been proposed in an effort to explain the processes leading to high TSH levels in individuals with high BMI, including variation in the activity of peripheral deiodinases leading to possible changes in thyroid hormone action at the cellular level (21). Some studies have shown that thyroid function may vary during overfeeding or starvation. This may represent a physiologic adaptation, causing changes in metabolism and energy expenditure that may affect the ability to control weight gain during overfeeding and weight loss during starvation (1,13,20,22,23,24,25,26). Leptin has been proposed as a possible factor that causes higher TSH levels. Leptin regulates both prothyrotropin-releasing hormone (pro-TRH) synthesis and TRH expression (27). TSH can directly stimulate leptin secretion by adipocytes (28). Furthermore, the presence of TSH receptors in adipose tissue (29,30) causes proliferation of adipose tissue and differentiation of preadipocytes into adipocytes (31,32). Higher levels of leptin are proportional to overall fat percentage (33), and some studies have shown a positive correlation with TSH levels (34,35,36).

The high serum TSH levels in individuals with high BMI could be due to the decreased expression and down regulation of the TSH receptor gene, which is less expressed in obesity. Consequently, the plasma TSH increases to deal with peripheral hormone resistance (37). Furthermore, thyroxine controls the metabolic rate, and small changes in the LT4 dose in patients with long-term LT4 treatment have been shown to modify resting energy expenditure and weight changes (38). Our patients had been on levothyroxine replacement for at least 6 months, and this could also explain the association between TSH levels and BMI.

### Study Limitations

A limitation of our study is that it was retrospective. The association between TSH and BMI and W/L percentiles could be affected by the presence of other modifying factors, such as different weight scales, diet, activity, emotional state, duration of treatment with levothyroxine, and compliance with medication.

## CONCLUSION

A significant positive association was observed between the serum TSH concentration and BMI percentiles in hypothyroid children who are chemically euthyroid while on thyroid hormone replacement. In this population, whether variations in TSH and free T4 levels within the normal range can influence body weight or whether obesity per se can alter thyroid function cannot be stated at this time. We could speculate that in hypothyroid children who are on treatment with levothyroxine, aiming to keep the TSH in the lower half of the normal range will help them lose or maintain their weight. However, it is impossible to determine whether the higher TSH levels are the cause of the increased BMI or vice versa. Further studies are needed to assess the link between thyroid function and body weight.

We could associate the BMI and TSH levels with other parameters such as abdominal fat deposit measured by ultrasound scan, skin fold thickness, and waist and hip circumference, but since this study was retrospective, we did not have the data available.

## Figures and Tables

**Table 1:**
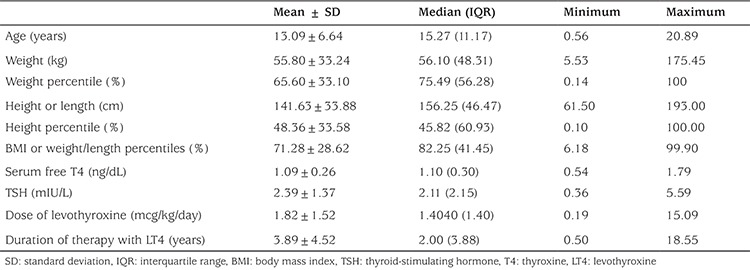
Anthropometric and hormonal parameters in the study subjects

**Table 2:**
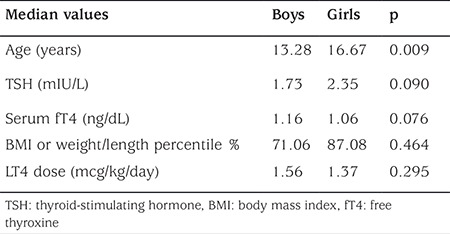
Comparison of median age, serum thyroid-stimulating hormone, serum fT4 levels, body mass index percentiles, and dose of LT4 between boys and girls

**Table 3:**
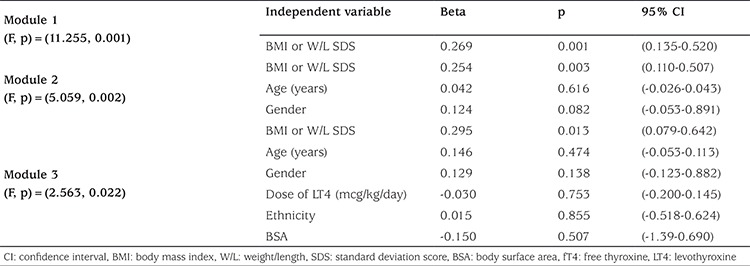
Simple and multivariate linear regression analysis with serum thyroid-stimulating hormone as dependent variable
